# Increasing Quantity and Internal Quality of Japanese Quail (*Coturnix coturnix japonica*) Eggs by Shooting Laser Puncture at Reproductive Acupuncture Points

**DOI:** 10.1155/2021/6621965

**Published:** 2021-03-22

**Authors:** Sunaryo Hadi Warsito, Tatang Santanu Adikara, Septiana Megasari, Ilham Radifan Pratama, Mirni Lamid, Herry Agoes Hermadi

**Affiliations:** ^1^Department of Animal Husbandry, Faculty of Veterinary Medicine, Universitas Airlangga, Surabaya, Indonesia; ^2^Department of Veterinary Anatomy, Faculty of Veterinary Medicine, Universitas Airlangga, Surabaya, Indonesia; ^3^Undergraduate Student of Veterinary Medicine, Faculty of Veterinary Medicine, Universitas Airlangga, Surabaya, Indonesia; ^4^Department of Veterinary Reproduction, Faculty of Veterinary Medicine, Universitas Airlangga, Surabaya, Indonesia

## Abstract

This study aims to determine the effect of laser puncture shooting on the reproductive acupuncture points of Japanese quail (*Coturnix coturnix japonica*) egg quantity (egg production) and internal egg quality (Haugh unit, yolk index, and yolk colour). This research was conducted for 30 days using a sample of Japanese quail aged 4 weeks because for the first 2 weeks, the quail did not produce and it was in production in the last 2 weeks. There were 4 treatments and 25 replications each. So, there were a total of 100 quails. Laser puncture shooting was carried out at 3-day intervals at the Ova point and 6-day intervals at Hu Men, Bei Ji, and Wei Gen points. So, on the first day, laser puncture shooting was carried out at 4 points, on the 4th day, only at 1 point, and then, on the 7th day, it returned to 4 points. And so, it was carried out for 4 weeks. T0 (−) was considered as a negative control because quails are not given laser puncture shooting; T0 (+) was considered as a positive control because quails are treated with laser puncture shooting which is deactivated or a dose of 0 Joule; T1 was a group treated with laser puncture shooting at a dose of 0.2 Joule, and T2 was a group treated with laser puncture shooting at a dose of 0.5 Joule. Then, the research results were analyzed using Analysis of Variance and followed by Duncan's Multiple Range Test. Laser puncture shooting at the reproductive acupuncture point of Japanese quail (*Coturnix coturnix japonica*) can increase the quantity of eggs (*p* < 0.05). While the results of the egg internal quality in the form of the Haugh unit value and the yolk index increased (*p* < 0.05), the value of the yolk colour did not change compared to the control (*p* > 0.05). Laser puncture shooting at the reproductive acupuncture point of Japanese quail (*Coturnix coturnix japonica*) can increase egg quantity (egg production) and internal egg quality (Haugh unit and yolk index) with the best dose of 0.5 Joule.

## 1. Introduction

Japanese quail is one of the poultry commodities from the genus *Coturnix* that can be used to produce eggs and meat. Japanese quail egg production can reach 200–250 eggs/year with an average weight of 10 g/eggs and adult female body weight 120–160 grams and adult male 100–140 grams, and they can be reared up to 2–2.5 years [1]. The advantages possessed by quails are as follows: high egg production, maintenance cages do not require a large place, dirt is not too smelly, and a short rearing period [2, 3].

Quail eggs are the best source of protein and fat. The nutritional value of quail eggs is not inferior to chicken eggs. Every 100 grams of quail eggs contain 12.7% of protein and 9.89% of fat. The most important amino acid found in the egg white is leucine, and the yolk contains the highest essential fatty acid, linoleic acid, and the highest nonessential fatty acid, oleic acid. In addition, it also contains vitamin E which is high in egg yolk and the sex hormone progesterone in the white and egg yolk [4, 5].

Indonesia, in general, has an average daily temperature above 30°C, except for the area around the mountains. This is very influential on the level of quail egg production. These conditions are consistent with research showing [6] that the production of quail egg at high environmental temperatures (31–34°C) is 10.77% lower than that at low environmental temperatures (26–30°C). Therefore, we need a solution to increase quail egg production. In addition, it is also necessary to have an added value for people who consume quail eggs through improving the quality of the eggs. Egg quality is determined based on quality internally and externally. Egg internal quality includes the Haugh unit, yolk index, yolk ratio, albumen ratio, albumen index, and egg yolk colour [7, 8]. One alternative technology that needs to be tried to increase the egg quantity (egg production) and quality is the use of laser spray technology.

Laser puncture is a technology that utilizes shortwave radiation beams that are fired at receptors (acupuncture points in livestock) aimed at increasing the capacity and efficiency of their organs so as to increase livestock productivity. Laser puncture technology also functions as a biological receptor that has links with related organs that can provide stimulatory, radiation, and inhibitory effects [9]. According to Whittaker [10], laser puncture is the shooting of traditional acupuncture points using nonthermal laser radiation with low intensity, and the most commonly used laser instrument is the semiconductor. The use of lasers in livestock aims to improve the biological balance and health of livestock; besides, the most important thing is to increase the ability of livestock productivity and improve the reproductive ability of livestock. To achieve the abovementioned goal, the laser is fired at the points associated with organs that function for health, balance, production, and reproduction in livestock [11].

The advantage of the laser method is that it can provide results that are easily controlled precisely and more accurately [12]. The use of low-power lasers to stimulate acupuncture points is fast, simple, and harmless at each point [13], so this technology is relatively cheaper because one device can be used en masse and with low operating costs rather than using acupuncture through a needle because the time can be adjusted according to the desired dose, so that, in 1 minute, at least 6 birds can undergo laser puncture. According to Ng et al. [14], acupuncture treatment can effectively overcome reproductive problems and be free from side effects.

This study aims to determine the effect of laser puncture shooting on the reproductive acupuncture points of Japanese quail (*Coturnix coturnix japonica*) on egg quantity (egg production) and internal egg quality (Haugh unit, yolk index, and yolk colour).

## 2. Materials and Methods

This research was carried out in the experimental animal enclosure of the Faculty of Veterinary Medicine, Universitas Airlangga, Surabaya, East Java, Indonesia, using 100 heads of Japanese quail (*Coturnix coturnix japonica*) in the growth phase which were 4 weeks old and reared in a wooden battery cage. Quails are fed commercially according to the age of the 4-week-old laying quail (growth phase), so that it has entered the spawning period. Drinking water is provided at all times (ad libitum). Lighting by the lamp is given during the 16–17 hour in layer phase. The laser puncture used is a semiconductor laser puncture 20–100 mW (milli Watt). Furthermore, the laser puncture is set to 100 mW of power, so that if wishing to get a dose of 0.2 Joule, we need to fire it for 2 seconds and for 5 seconds to get a dose of 0.5 Joule.

**Figure 1:**
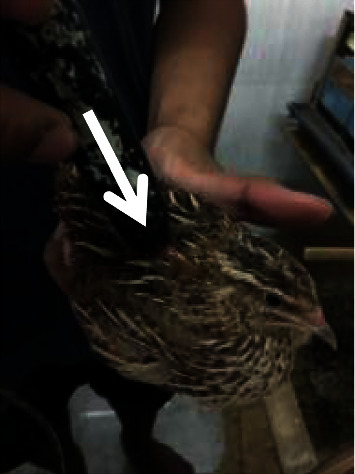
Laser puncture shooting at the Ova point.

**Figure 2:**
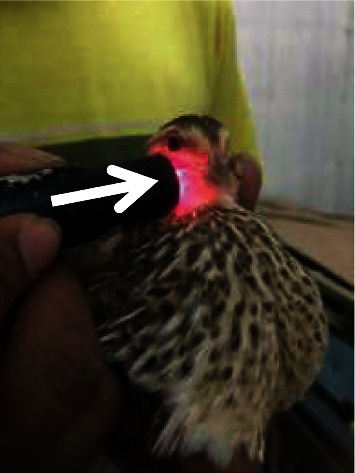
Laser puncture shooting at the Hu Men point.

**Figure 3:**
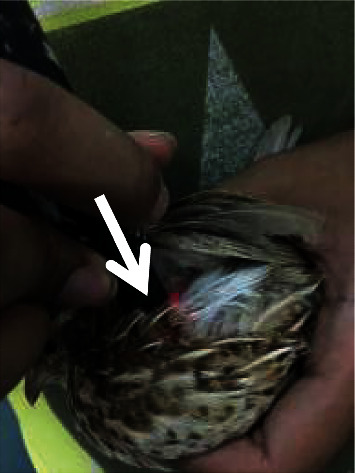
Laser puncture shooting at the Bei Ji point.

**Figure 4:**
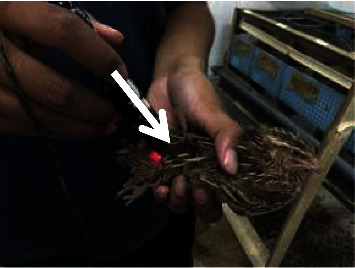
Laser puncture shooting at the Wei Gen point.

Quails are handled in an upright position as comfortable as possible so that the quails do not move. Laser puncture shooting is given at the Ova point (Figure 1) as a reproductive point located in the dorsal side of the last thorax and first lumbar joint once every 3 days, and laser puncture shooting was performed at 3 additional points at 6-day intervals, namely, the Hu Men point (Figure 2) located at the beak angle next to the caudoventral angle the mouth of the dexter and sinister, the Bei Ji point (Figure 3) located at the base of the apex axillary fossa dexter and sinister, and the Wei Gen point (Figure 4) located at the sacrococcygea joint. After finding the right stimulation points, shooting using a laser beam is carried out. The laser beam is fired using doses of 0, 0.2, and 0.5 Joule. The shooting is carried out once at that point. The machine will automatically turn off if the dose that has been fired is as desired. The shooting was carried out on all treatments of T0 (+), T1, and T2, whereas T (−) is a control group that was not given a laser puncture shooting treatment.

The research design carried out in this study is a completely randomized design with 4 treatments each of 25 repetitions so that there are as many as 100 female quail samples. Quails were randomly divided into 4 groups, namely, T0 (−) as a negative control because quails were not given laser puncture shooting; T0 (+) as a positive control because quails were treated with laser puncture shooting which was deactivated or a dose of 0 Joule; T1 as a group treated with laser puncture shooting at a dose of 0.2 Joule; and T2 as a group treated with laser puncture shooting with a dose of 0.5 Joule. Laser puncture shooting at the point of reproduction was carried out after adaptation for 8 days, namely, the 9^th^ to 30^th^ day with a time interval of 3 days at the Ova point and 6-day interval at the Hu Men, Bei Ji, and Wei Gen points.

Egg productivity is calculated by counting the number of eggs produced by quails per day, recorded, and then, added from days 9 to 30, so the quail egg production results obtained in the study were called quail day production. Furthermore, the internal quality of the eggs is checked by breaking the eggs and doing internal data collection of eggs, and this is carried out every day until the end of the research. The egg internal data obtained using calipers and micrometers were used to determine the value of the Haugh unit and the yolk index, and an egg yolk colour fan was used to assess the colour of the yolk.

Furthermore, the results of the study were analyzed by using Analysis of Variance followed by Duncan's Multiple Range Test (Table 1).

## 3. Results

### 3.1. Egg Quantity

Quail egg production results obtained from days 9 to 30 are referred to as quail day production, as shown in Table 2. The results showed that, between controls T (−) and T (+), there were no significant differences between treatments (*p* > 0.05). However, between the control treatment and laser puncture shooting, there were significant differences (*p* < 0.05), while there were no significant differences between the treatments given by laser puncture shooting doses of 0.2 and 0.5 Joule (*p* > 0.05).

### 3.2. Internal Quality of Eggs

The results of research on the internal quality of eggs include examining the value of the Haugh unit (Table 3), the yolk index (Table 4), and the yolk colour (Table 5). In the examination of the Haugh unit value, the results show that, in control T (−) and T (+), there is no significant difference between treatments (*p* > 0.05) and also not significantly different from T1. While there was no significant difference between T1 and T2 between treatments (*p* > 0.05), between T2 with T0 (−) and also T0 (+) control, there were significant differences between treatments (*p* < 0.05).

On examination of the yolk index values in the obtained results in the control T (−) and T (+), there were no significant differences between treatments (*p* > 0.05), while between T1 and T2, there were significant differences between treatments (*p* < 0.05), as well as between T1 and T0 (−) and T0 (+) and T2 with T0 (−) and T0 (+), there were significant differences between treatments (*p* < 0.05).

In the examination of the yolk colour results obtained in all treatments, both in the control T (−) and T (+) and also T1 and T2, there were no significant differences between treatments (*p* > 0.05). So, laser puncture shooting does not have a significant effect on the yolk colour value.

## 4. Discussion

### 4.1. Egg Quantity

Quail day production (QDP) is the highest in quail eggs in T2 with 62.50% by laser puncture shooting at a dose of 0.5 Joule but not significantly different from that in T1, i.e., 59.09%, and the lowest in T0 (+) with 48.29% which were shot at a dose of 0 Joule and also T0 (−) without laser puncture shooting treatment, which is 49.17%. In this study, the reason for the presence of a negative control T0 (−) and a positive control T0 (+) is to find out whether there is an effect (stress) on the quails because they are not held or T0 (−) and some are held but with a dose of 0 Joule or T0 (+). Based on the research results, it turns out that the shooting of the laser puncture can directly affect the target organs as a whole, so an increase in the capacity and efficiency of the organs can be achieved which is described in the form of increased “Biological Achievement,” namely, increased production. It is called a biological achievement because it is strongly suspected that there is a change in the reproductive organs after shooting of the laser puncture was 2 weeks before the quail production time and 2 weeks while it was in production. This is indicated by a significantly different QDP (*p* < 0.05) with the results of T1 (0.1 Joule) and T2 (0.5 Joule) being more elevated.

According to Abdurachman et al. [16], the use of laser puncture with a dose of 0.1 to 0.5 Joule will provide a stimulating effect that is an increase in energy at the acupuncture point and an effect on the physiological capacity of the target organ, while doses above 0.5 Joule will provide a sedation effect, i.e., decreased energy or calming down on excessive organ performance so that an energy balance occurs in accordance with the condition of the organ.

The shooting of laser puncture at the reproductive acupuncture point with a dose of 0.2 Joule and 0.5 Joule in this study proved to be able to increase the quail day production (QDP) of quails, but the QDP of quails shot by a 0.2 Joule laser puncture dose did not make a significant difference with QDP quail shot by a 0.5 Joule laser puncture dose. This shows that the lowest dose of treatment in laser puncture studies has been effective in increasing the QDP of quails. At a dose of 0.5 Joule, it may be the beginning of the effect of sedation, so the increase in egg production is not significant or is optimal.

Increased egg production due to cells at the acupuncture point has different electrical properties from the surroundings. This causes cells to be more sensitive to stimuli and able to deliver these stimuli through cellular systems in the body of living things (in a biological process) [17]. Stimulation at the reproductive acupuncture point (Ova point) can increase ovarian activity (estrogen hormone). The Ova point is analogous to the GV-4 (Ming-Men) point in humans [18].

The mechanism of increased egg production due to laser puncture stimulation at the Ova point is supported by shooting at the Hu Men and Bei Ji points. Stimulation at the Ova point gives rise to energy. The energy enters from the bone marrow to the central nervous system (CNS), so the energy is channeled to the hypothalamus. The stimulation at the acupuncture point is able to optimize the production of GnRH (Gonadotropin-Releasing Hormone) because the laser functions to meet the feed ingredients in the body, so that the organs can function optimally and efficiently. The laser supports the hypothalamic process of a GnRH clot caused by stimulation from light or light from a lamp in a cage. Light from a lamp can stimulate the hypothalamus to synthesize and secrete GnRH, both through the hypothalamic retino pathway or direct penetration in cranial bones and brain tissue [19].

The hypothalamus has an important role in the regulation of LH (Luteinizing Hormone) secretion through positive feedback related to the production of progesterone from the largest ovarian follicle in the follicle hierarchy (number of eggs). The development of follicles will initiate estrogen secretion in external theca cells and progesterone by granulosa cells. Growing prehierarchic follicles secrete estrogen in low concentrations. With the development of prehierarchic follicles into hierarchical follicles, estrogen secreted is increasing. Estrogen has the effect of increasing levels of calcium, protein, fat, vitamins, and other substances in the blood needed for egg formation. Increasing the size of the follicles in the follicular hierarchy will reduce estrogen secretion and increase progesterone synthesis. Progesterone is synthesized by preovulation follicles which can stimulate the anterior pituitary to secrete LH (Luteinizing Hormone), so progesterone secreted into the blood is a positive feedback for LH secretion from the anterior pituitary. LH increases will cause ovulation, namely, the release of yolk that has been cooked from the ovaries into the infudibulum. The speed of adult female genital poultry is characterized by the first ovulation process [20].

Egg production is affected by feed. Hu Men and Bei Ji points support efficient feed, so egg production can increase. The Hu Men point increases appetite, digestibility, and absorption in quails. This is confirmed by research [18] that stimulation at the Hu Men point can provide electrical and ionic changes in the area, so membrane depolarization occurs. Membrane depolarization causes an action potential or causes an electric current to be sent to the brain to stimulate the hunger center. The mechanism of action of acupuncture in overcoming eating disorders is one of them through improvement of gastric emptying. Increased gastric and intestinal emptying time is associated with increased hunger. Stimulation at several acupuncture points has been shown to improve gastrointestinal motility, so increased gastric motility will increase the time of emptying the stomach and will improve the appetite.

Stimulation at the Bei Ji point is very influential on improving lung performance and absorbing more oxygen, and the heart will supply oxygen to tissues by increasing cardiac output [18]. Oxygen is used by the mitochondria to produce adenosine triphosphate (ATP), a compound that is a source of energy through oxidative phosphorylation that occurs in the mitochondria. The core of the process of oxygen metabolism is food that enters the body will undergo an oxidation process by releasing electrons which will then be received by electron carriers such as nicotinamide adenine dinucleotide (NAD+) and flavin mononucleotide (FAD). Oxidation is a reaction in which electrons are moved from one atom to another [21]. Nicotinamide adenine dinucleotide (NADH) and flavin (FMNH2 and FADH2) are reduced to be oxidized again by oxygen in the mitochondria, so that a large amount of ATP will be produced. The whole process takes place in the inner mitochondria, ATP functions as an energy storage, and ATP will be released little by little to drive the chemical reactions needed by cells [22]. Laser puncture shooting at the Wei Gen point in research is intended to keep quails resistant to disease. Stimulation at the Wei Gen point can increase antibodies humorally and cellularly so that it can increase endurance [18], so in research, it is apparent that laser puncture can increase the capacity and efficiency of the function of the reproductive organs, digestion, and endurance of the body in supporting increased egg production.

### 4.2. Egg Quality

The Haugh unit is albumen quality measured by albumen height and egg weight. The higher the value of the Haugh unit, the better the egg quality. The Haugh unit value is influenced by gene, age, season, egg, and feed storage conditions [23–25]. The results showed that the shooting of laser puncture with a dose of 0.2 Joule was able to increase the Haugh unit value of quail eggs, whereas the dose between 0.2 and 0.5 Joule did not show a significant difference (*p* > 0.05).

The high value of the yolk index is influenced by the protein and fat content in the feed which functions as a mediator in the process of egg formation [26]. The function of protein and fat is to maintain egg viscosity, especially in the chalaza, which helps the yolk remain in the middle and also forms a vitelin membrane or a strong egg yolk wrapper [27]. The results of the research showed that laser puncture shooting at a dose of 0.2 Joule was able to increase the quail yolk index value, but at a dose of 0.05 Joule gave the best results when compared with a dose of 0.2 Joule, especially with the two control treatments, i.e., T (−) and T (+).

According to Zaheer [28], the yolk colour score is also influenced by nutrient content in the feed, for example, beta-carotene and xanthophyll in the feed. Beta-carotene is a carotenoid group compound that is unstable because it is easily oxidized to xanthophyll which functions as an egg yolk dye. The results of the study showed that laser puncture shooting with doses of 0.2 and 0.5 Joule was not able to increase the yolk colour value of the quail eggs. So, it can be said that laser puncture shooting cannot change the yolk colour of quail eggs.

The body's digestible feed will be absorbed for the animal's metabolic needs. Protein in feed can be absorbed maximally into the digestive system and distributed to eggs, especially in egg whites. The protein is synthesized further before ovulation and increases when the egg is inside the magnum. Yolk in the oviduct is a signal for magnum epithelial cells to synthesize and secrete the egg white component into the magnum lumen. In this study, it was shown that laser puncture shooting conducted at the Hu Men Point/ST-4 increased appetite and drinking, as well as increased digestive organ activity, among others, and it is suspected that the increase in digestibility and absorption of feed, in general, is due to the increased level of production as mentioned in the previous discussion. However, the impression is not able to increase the absorption of beta-carotene and xanthophyll in the feed. This is thought to be possible because the two nutrients have been absorbed properly, so that even if laser puncture is performed, the yellow colour will still not change.

Quails have a high level of stress on the surrounding environment. Laser puncture treatment right at the poultry acupuncture point can stimulate the bird to be more comfortable and relieve stress. The Wei Gen/GV-2 point (a poultry body endurance point), Bei ji point/HT-1, BL-13, and BL-15 stimulate the working of the heart and lungs including increasing blood flow throughout the body and increasing oxygen consumption. The Ova point is analogous to the GV-4 (Ming-Men) point in humans [18]. The Ova Point is a poultry reproduction system that can optimize the performance of poultry reproductive organs.

Adikara [18] further stated that the mechanism of semiconductor laser puncture shooting on the acupuncture points produces energy and travels through the acupuncture meridians to target organs, after which physiological and biochemical changes occur. Physiological and biochemical changes include enzyme synthesis, hormonal system, and changes in performance with organs in accordance with the laser dose given, i.e., the dose for stimulation or the dose for sedation. Biophysical stimulation will produce energy that is flowed into a body's meridian system and other systems. The way laser puncture works the same as acupuncture which is to shoot the laser at the acupuncture point will have an effect on the excitation site and in a place far from the excitation site through nerve pathways (peripheral and central nerves), neurohumoral and meredian. The related organ will respond to the energy it receives, and there will be physiological and biochemical changes, such as increased work and organ capacity and enzyme or hormonal formation.

## 5. Conclusions

In general, the results of this study can be concluded that laser puncture shooting at the point of reproduction (Ova) which was added to the points of Bei ji, Wei Gen, and Hu Men in Japanese quail (*Coturnix coturnix japonica*) can increase egg quantity (egg production) and internal egg quality (especially the Haugh unit and yolk index, except egg yolk colour) because there were significant differences compared to the control treatment. The best laser puncture shooting dose recommendation is 0.5 Joule.

## Figures and Tables

**Table 1:** Chemical composition and energy of the quail diet.

Crude protein	21%
Metabolic energy	2900 kcal/kg
Crude fat	5%
Crude fiber	5%
Ash	7%
Calcium	3%
Phosphor	0.7%
Lysine	1%
Methionine	0.5%
Methionine + cystine	0.65%

Source: own formulation based on the Indonesian National Standard (SNI 01-3907-2006) [15].

**Table 2:** Average of quail day production in quails (*Coturnix coturnix japonica*).

Treatment	Average of quail day production ± standard deviation (%)
T0 (−)	49.17^a^ ± 5.79
T0 (+)	48.29^a^ ± 5.40
T1	59.09^b^ ± 6.87
T2	62.50^b^ ± 7.58

Note: different superscripts show a significant difference (*p* < 0.05).

**Table 3:** Average of the Haugh unit in quails (*Coturnix coturnix japonica*).

Treatment	Average of the Haugh unit ± standard deviation
T0 (−)	72.635^a^ ± 2.181
T0 (+)	73.275^a^ ± 2.953
T1	74.983^ab^ ± 3.253
T2	77.320^b^ ± 4.206

Note: different superscripts show a significant difference (*p* < 0.05).

**Table 4:** Average of the yolk index in quails (*Coturnix coturnix japonica*).

Treatment	Average of the yolk index ± standard deviation
T0 (−)	0.329^a^ ± 0.018
T0 (+)	0.334^a^ ± 0.013
T1	0.363^b^ ± 0.016
T2	0.383^c^ ± 0.027

Note: different superscripts show a significant difference (*p* < 0.05).

**Table 5:** Average of yolk colour in quails (*Coturnix coturnix japonica*).

Treatments	Average of yolk colour ± standard deviation
T0 (−)	6.214^a^ ± 0.426
T0 (+)	6.000^a^ ± 0.000
T1	6.143^a^ ± 0.363
T2	6.214^a^ ± 0.579

Note: the same superscript shows no significant difference (*p* > 0.05).

## Data Availability

The dataset used and analyzed during this study is available from the results of treatment of quails in experimental animal cages. The datasets generated and/or analyzed during the study are not currently publicly available, but are available from the respective authors upon reasonable request.
